# Commissioning of a novel gantry-less proton therapy system

**DOI:** 10.3389/fonc.2024.1417393

**Published:** 2024-11-07

**Authors:** Jon Feldman, Alexander Pryanichnikov, Alejandro Achkienasi, Ilya Polyansky, Yair Hillman, Stas Raskin, Philip Blumenfeld, Aron Popovtzer, Michael Marash

**Affiliations:** ^1^ Sharett Institute of Oncology, Hadassah Medical Center, Hebrew University of Jerusalem, Jerusalem, Israel; ^2^ P-Cure Ltd./Inc, Shilat, Israel; ^3^ Division of Biomedical Physics in Radiation Oncology, German Cancer Research Center (DKFZ), Heidelberg, Germany

**Keywords:** intensity modulated proton therapy, pencil beam scanning, synchrotron, gantry-less patient positioning, commissioning, calibration, dosimetry, quality assurance

## Abstract

**Purpose:**

The focus of this article is to describe the configuration, testing, and commissioning of a novel gantry-less synchrotron-based proton therapy (PT) facility.

**Materials and methods:**

The described PT system delivers protons with a water equivalent range between 4 and 38 cm in 1800 energy layers. The fixed beam delivery permits a maximum field size of 28 × 30 cm^2^. The patient positioning and imaging system includes a six-degree-of-freedom robotic arm, a convertible patient chair, a vertical 4DCT, and an orthogonal 2D X-ray imaging system.

**Results:**

The spot positioning reproducibility was consistent within ±1 mm. The width (σ) of the beam profile at the isocenter was energy dependent and ranged from 2.8 mm to 7.7 mm. Absolute dose reproducibility was measured and deviations were found to be <0.62% for all possible beam scenarios. The built-in dose monitoring system was successfully tested for its ability to generate interlocks under specific conditions (beam spot deviation ≥2 mm, individual spot dose ≥10% or ≥0.25 Gy, spot energy deviation ≥0.5 MeV). The robot positioning exhibited a consistent reproducibility within ±1 mm. All tested scenarios achieved laser-free initial 3D/3D image-guided positioning within ±5 mm. Subsequent 2D/3D positioning showed an accuracy of ±1 mm. A single 2D/3D image registration event corrected positions in all cases. Results of gamma analysis (3%, 3 mm) demonstrated pass rates greater than 95% for head and neck, thorax, abdomen treatment plans.

**Conclusions:**

We report on the performance of a novel single-room gantry-less PT system comprised of a compact synchrotron and an adjustable (from nearly horizontal to almost vertical) patient positioning system. The commissioning results show high accuracy and reproducibility of the main proton beam parameters and the patient positioning system. The new PT facility started patient treatments in March 2023, which were the first in Israel and the Middle Eastern region.

## 1 Introduction

Proton therapy (PT) has been recognized as one of a preferred cancer radiation treatment modality in recent years (1). In many cases two or three proton fields can offer a better dose distribution than X-ray intensity-modulated and volumetric modulated arc radiation therapy (2) due to their characteristic depth-dose distribution including finite range and the Bragg peak (3). Clinical trials have confirmed the effectiveness of PT in treating a variety of cancers (4–11), especially for pediatric tumors (12, 13). PT is becoming more widely used (14), with approximately 130 facilities in operation (15) and many more in the planning or construction stages.

Unfortunately, the important physical advantages and often proven clinical efficacy in most cases coincide with a high capital investment required to build and maintain a contemporary proton treatment center (16). One possibility to reduce the construction and maintenance costs of a PT center is to consider a compact variable energy proton synchrotron, replacing isochronous cyclotrons or synchrocyclotrons. Modern synchrotrons dedicated to proton therapy have a lower weight (15 tons) (17) than existing cyclotrons (240 tons for IBA C230, 90 tons for Varian Probeam) and synchrocyclotrons (46 tons for IBA S2C2, 22 tons Mevion S250) (18, 19). They can deliver multiple energies directly without needing beam degraders or collimators, resulting in a better quality of proton beams, a lower background radiation and, consequently, more compact facilities with less shielding requirements. This enables the facility to be located closer to the treatment rooms (20) and thus reduces the footprint of the PT center.

Another possibility to decrease costs is to treat patients in a rotating upright position instead of using an expensive rotating proton gantry (21). Until recently, standard upright treatment was combined with imaging in the supine position, raising concerns about potential differences in organ and tumor positioning between these two setups, limiting the widespread adoption of upright proton therapy. With the advent of vertical CT systems (22, 23) and upright treatment positioning systems are generating a renewed interest in this topic (24–26). New positioning and motion mitigation techniques for upright and semi-upright treatment (27–29) have been developed along with novel treatment chair designs (30, 31). These developments made low-cost solutions for PT more realistic.

The aim of this paper is to describe the commissioning and implementation of a new cost-efficient PT system that combines a modern compact synchrotron and an innovative adjustable (nearly horizontal to upright) gantry-less patient positioning.

## 2 Materials and methods

### 2.1 Accelerator

**Figure 1:**
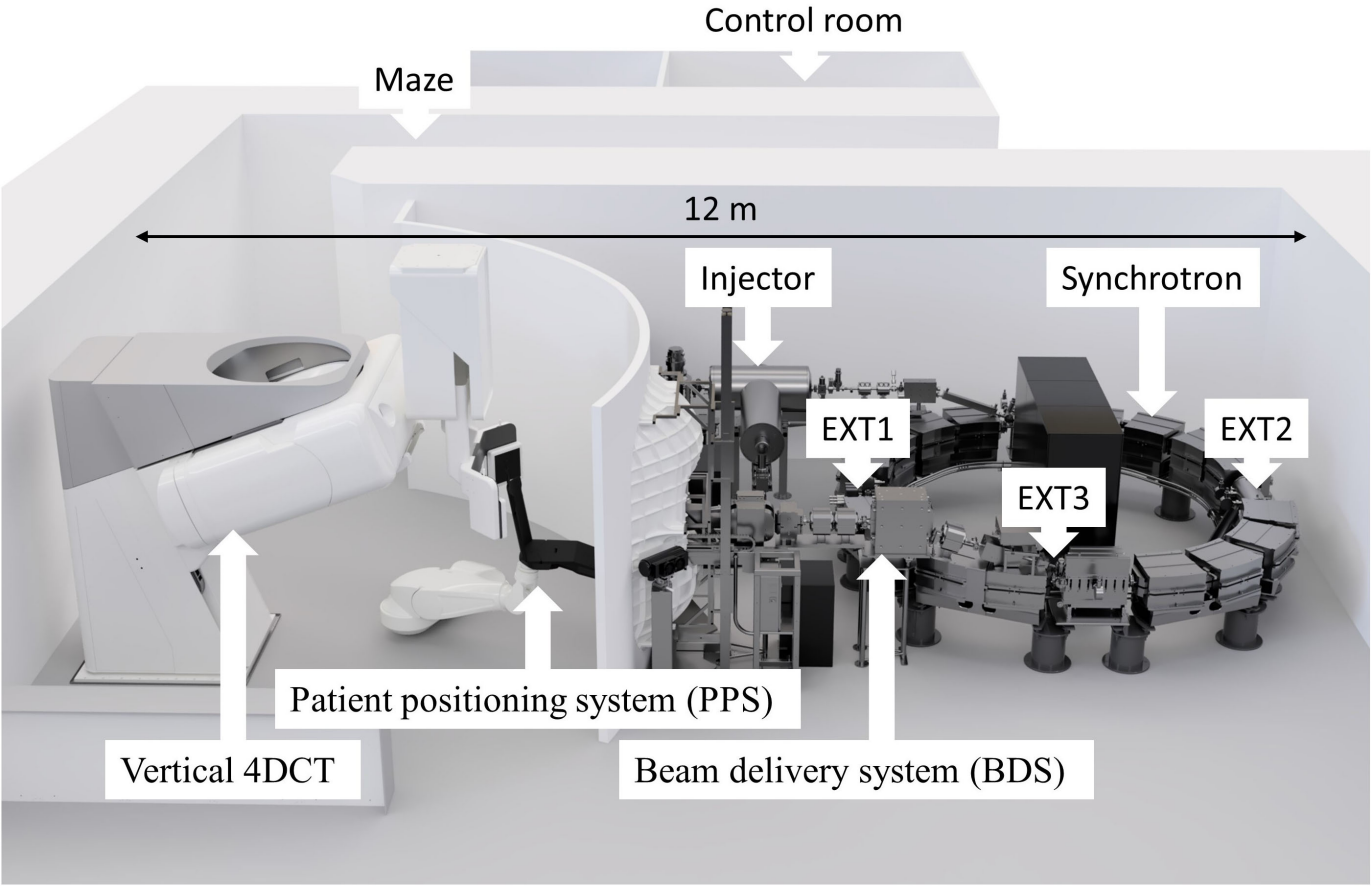
A novel proton therapy facility in Shilat, Israel with key components labeled, where EXT1 - extraction target station, EXT2 - extraction electrostatic deflector, EXT3 - extraction channel. The total footprint required for the system is 12 × 7.5 m, including patient positioning and imaging system.

The accelerator of the new P-Cure proton therapy facility in Shilat, Israel (**Figure 1**) is a modified version of the Prometheus compact proton synchrotron (17, 32). Like the original Prometheus system (JSC Protom, Protvino, Russia), the P-Cure medical accelerator system comprises an H^-^ ion source, a tandem accelerator as an injector, the main synchrotron, and a proton beam extraction and transfer channel. The principle of acceleration is as follows: on demand, the ion source delivers singly negatively charged hydrogen ions (H^-^) to the tandem accelerator, where the ions are stripped, converted into protons, and accelerated to the injection energy. The synchrotron then captures the injected protons and accelerates them up to the selected energy in the clinical range of 70-250 MeV with a precision of 0.1 MeV. The whole process of proton generation and acceleration to the maximum energy takes about 900 ms. After acceleration, particles can be extracted from the synchrotron for 5 ms to several seconds, depending on the number of spots and beam intensity. The synchrotron uses a slow resonant multiturn extraction scheme with modified orbit positioning. The orbit change is performed by the dynamic operation of 16 horizontal electromagnetic correctors located in each dipole magnet of the synchrotron. As a result of the distortion of the beam trajectory at the moment of extraction, the particles interact with one of the four beryllium targets (installed in the target gap inside the synchrotron, **Figure 1**, EXT1) and lose some energy. Due to this energy loss, the protons pass the electrostatic deflector (located at the smaller radius compared to equilibrium orbit, **Figure 1**, EXT2) and are directed into the extraction channel of the accelerator (**Figure 1**, EXT3). The extraction rate is determined by the parameters of the excitation frequency, the beryllium target and the orbital position.

The key difference between this accelerator and the original Prometheus system is the novel design of the injection system. The injection energy has been increased to 1.1 MeV compared to the previous design with 0.9 MeV energy, and the recharge target inside the tandem accelerator has been changed from a carbon film to a nitrogen jet which increases the beam intensity in the synchrotron ring. This change also has the advantage of removing the destructible carbon film, which needs to be replaced regularly (32).

The accelerator is among the most compact solutions for proton production in the clinical energy range. The synchrotron’s outer diameter is 5 meters, and the equipment weighs approximately 15 tons. Another significant advantage of this system is its very low average power consumption of about 20 kW in stand-by mode (no acceleration) and about 110 kW for the extraction beam at maximum energy.

The accelerator control system is fully digitized and driven by a combination of software and hardware interfaces that are used to tune subcomponents and provide real-time feedback for monitoring and adjustment. Central to the system is the accelerator server, which collects and presents data from an independent beam monitoring system. Clinical settings, produced by imaging and planning systems, are pre-processed, and transferred to the client controller. These settings are then sent to the accelerator server, where the power supplies and radiofrequency parameters are configured.

### 2.2 Beam delivery system

**Figure 2:**
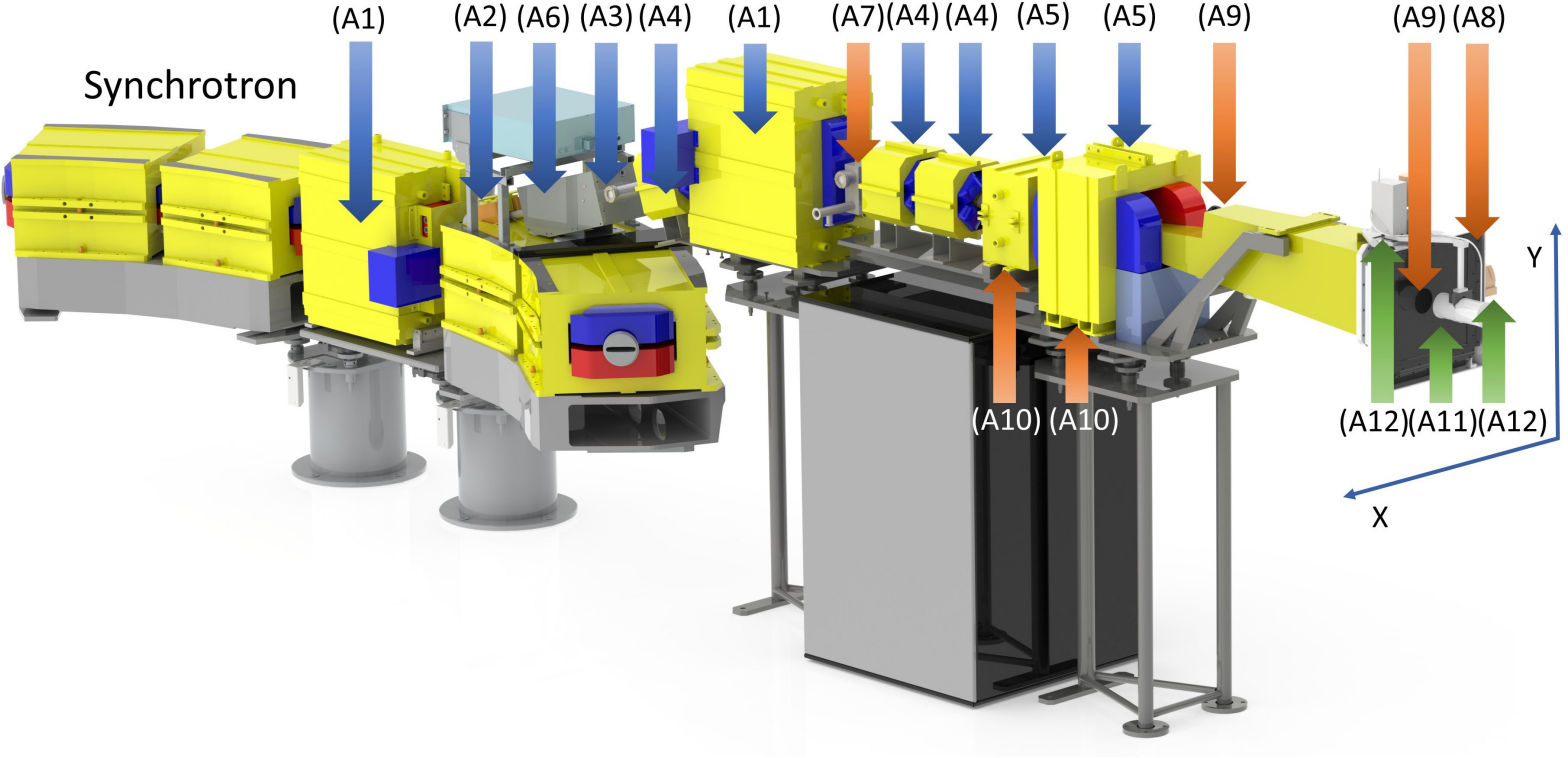
Components of the beam delivery system (BDS) and the dose monitoring system (DMS), indicated by arrows and numbered as described in the main text. The primary elements of the BDS, including parts of the magnetic optics and the beam control module, are indicated by blue arrows, the redundant beam measurements provided by the DMS are indicated by orange arrows, and the removable external elements are indicated in green.

The synchrotron proton beam is extracted during a predetermined beam gate into the beam delivery system (BDS) shown in **Figure 2**. Primary components of the BDS are marked with blue arrows and external devices are marked with green arrows. The number labels A1 - A4 in **Figure 2** correspond to the individual BDS components as described below. The BDS is responsible for beam transporting and assigning appropriate pencil beam parameters (X and Y sigma and coordinates) as defined by the treatment plan. It consists of two bending magnets equipped with vertical electromagnetic correctors (A1); a horizontal electromagnetic corrector (A2); a beam control module (BCM) containing a thin (about 20 µm) scintillator film (inside vacuum) and a photomultiplier (outside vacuum) (A3); focusing quadrupoles (A4); scanning magnets in the horizontal and vertical directions, (A5); a fast magnetic shutter (FMS), (A6); a dose monitoring system (DMS), (A7-A10) described separately below; a Faraday cup, (A11); lasers, (A12); and an external range shifter to reduce the beam range below 4 cm and adjust dose distributions according to treatment planning scenarios including irradiation of superficial (≤ 3 cm depth) tumors (not shown in **Figure 2**).

The BDS-associated BCM provides feedback on the number of protons passing through the scintillator film (A3). It is calibrated by an external removable Faraday cup (A11) prior to the start of each 8-hour shift. The FMS provides rapid termination of the proton beam by magnetically diverting it away from the nominal extraction channel trajectory and stopping the acceleration cycle by providing a feedback signal to the accelerator control server. Activation of the FMS is triggered either by reaching the prescribed dose limit of a given spot/energy level for normal clinical operation, or because of a system interlock due to a signal from the facility’s internal safety system. The magnet of the FMS (“on” by default) generates a magnetic flux density of up to 0.12 T. The FMS is capable to divert the beam within 50 µs.

The maximum field size formed by the BDS at the irradiation isocenter is approximately 28 cm × 30 cm. The isocenter is located at the distance of 700 mm from the edge of the nozzle. Vertical and horizontal lasers (A12) define the irradiation isocenter. In the commissioning process, laser positions were calibrated using the Lynx (IBA Dosimetry, Schwarzenbruck, Germany) scintillation detector at the irradiation isocenter, employing a 250 MeV beam with the smallest transverse size and a calibration pulse of 10^9^ protons. Beam positions for other energies were adjusted relative to this reference point.

### 2.3 Dose monitoring system

The novel Dose Monitoring System (DMS) was developed and implemented for the first time. It independently tracks the critical beam parameters with a time resolution of 200 µs. The DMS comprises two types of particle number counters, beam position detectors, and beam energy sensors that provide redundancy for these measurements. These beam parameter sensors are marked with orange arrows and are shown in **Figure 2**.

The first DMS-associated particle counter (A7) is a BCM that is located after the second bending magnet of the BDS. It consists of a 10 µm plastic film coated with single layer ~10 µm of ZnO and is read by a single photomultiplier. The BCM monitors the number of protons traversing with intensities up to 2×10^10^ protons/s for energies up to 330 MeV. The second DMS-associated particle number counter, IC128 (Pyramid Technical Consultants, Waltham, MA, USA), (A8), is an array of 128 ionization chambers spread over an active area of 25 cm × 25 cm and has a water-equivalent thickness (WET) of 200 µm. IC128 is optimized to measure proton current densities up to 30 nA/cm^2^ over an energy range of 30 – 500 MeV.

The first DMS-associated beam position detector (A9) uses a gadolinium-coated (P43-phosphor) vacuum window (~100 µm WET) in the nozzle to monitor the beam position with a fast (300 fps) video camera. The second DMS-associated beam position detector (A10) is a pair of current sensors that read the electrical currents through the horizontal and vertical scanning magnets (A5), respectively. These readings are calibrated using IBA Lynx as the external beam position detector for the full range of energies and scanning magnet temperatures.

The third function of the DMS is to provide two redundant measurements of the extracted beam energy. Specifically, the first of the two energy detectors measures the frequency of the accelerating station during the final milliseconds of the accelerator cycle leading up to beam extraction. The second energy detector calculates the frequency of beam revolution in the synchrotron by utilizing an inductive beam current sensor located in the first rectilinear section of the synchrotron following injection. Both measurements are converted to particle energy and corrected for the energy loss during the passage of four thin beryllium extraction targets.

The beam intensity, position, and energy for each planned beam spot along with its ID is sent to the DMS-associated fast microcontroller, which also stores the treatment planning data for each spot ID. Measured and stored data are compared, and a beam enable signal is sent to the FMS in case of agreement within tolerance. In case of an interlock, the FMS terminates the beam extraction within 50 µs, and the accelerator server sends a message to the client interface with the reason for the interlock.

### 2.4 Patient positioning and imaging system

**Figure 3:**
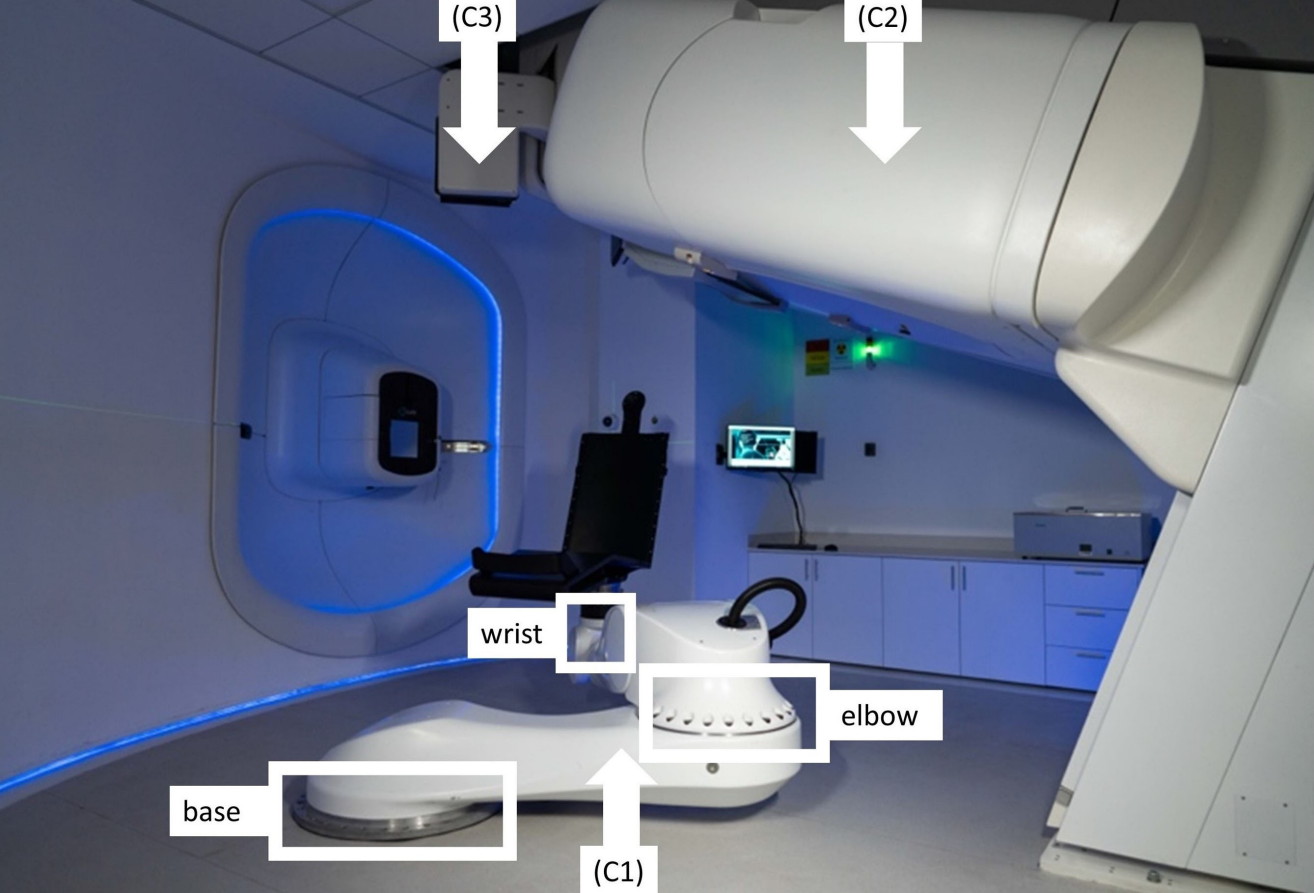
The P-ARTIS system consists of a patient positioning system (PPS) (C1) with mounted patient support (a chair); vertical 4DCT (C2) and X-ray orthogonal 2D system (C3).

The PAtient Robotic posiTioning and Imaging System (P-ARTIS) comprises a patient positioning system (PPS) with a convertible patient chair (C1), a vertical 4DCT imaging system (C2), an orthogonal 2D X-ray imaging system (C3) as shown in **Figure 3**, and control electronics and software.

The P-ARTIS PPS kinematics is based on the Leoni Orion (Leoni, Montigny-le-Bretonneux, France), a six-degree-of-freedom (DoF) robotic system approved for particle therapy applications (35). The robot’s translational motion is determined by three axes: two orthogonal axes (X, Y) depicted in **Figure 2**, and a Z-axis adjusted to be parallel to the beam trajectory. The maximum lateral travel distance is 4.880 m, vertical - 1.184 m, and longitudinal - 2.230 m. Its geometry (**Figure 3**) includes articulating surfaces: base, elbow, and wrist. The elbow/base allows left and right ±25° inflections, while the wrist offers ±100° rotation, enabling full 360° access to the treatment volume via elbow/base inflection. The PPS has been calibrated for various loads up to 180 kg with ±0.5 mm accuracy (95% confidence) and has the capacity to scale the weight up to 240 kg.

The P-ARTIS CT utilizes a Phillips Brilliance Big Bore platform angled at 20° relative to the vertical axis of the room. It retains the manufacturer’s operating characteristics, with modifications limited to motion control and reporting interfaces. Patient motion during image acquisition is managed by a sliding platform on the CT base, using the same control interface as the traditional moving couch. A respiratory motion kit facilitates 4D applications. The 2D X-ray system provides planar, orthogonal radiographic imaging of patient geometry at the treatment isocenter position. It is designed with two 150 kV X-ray sources positioned on either side of the BDS and ceiling-mounted retractable 30 cm × 30 cm flat PaxScan 3030DX detectors (Varian Medical Systems, USA).

**Figure 4:**
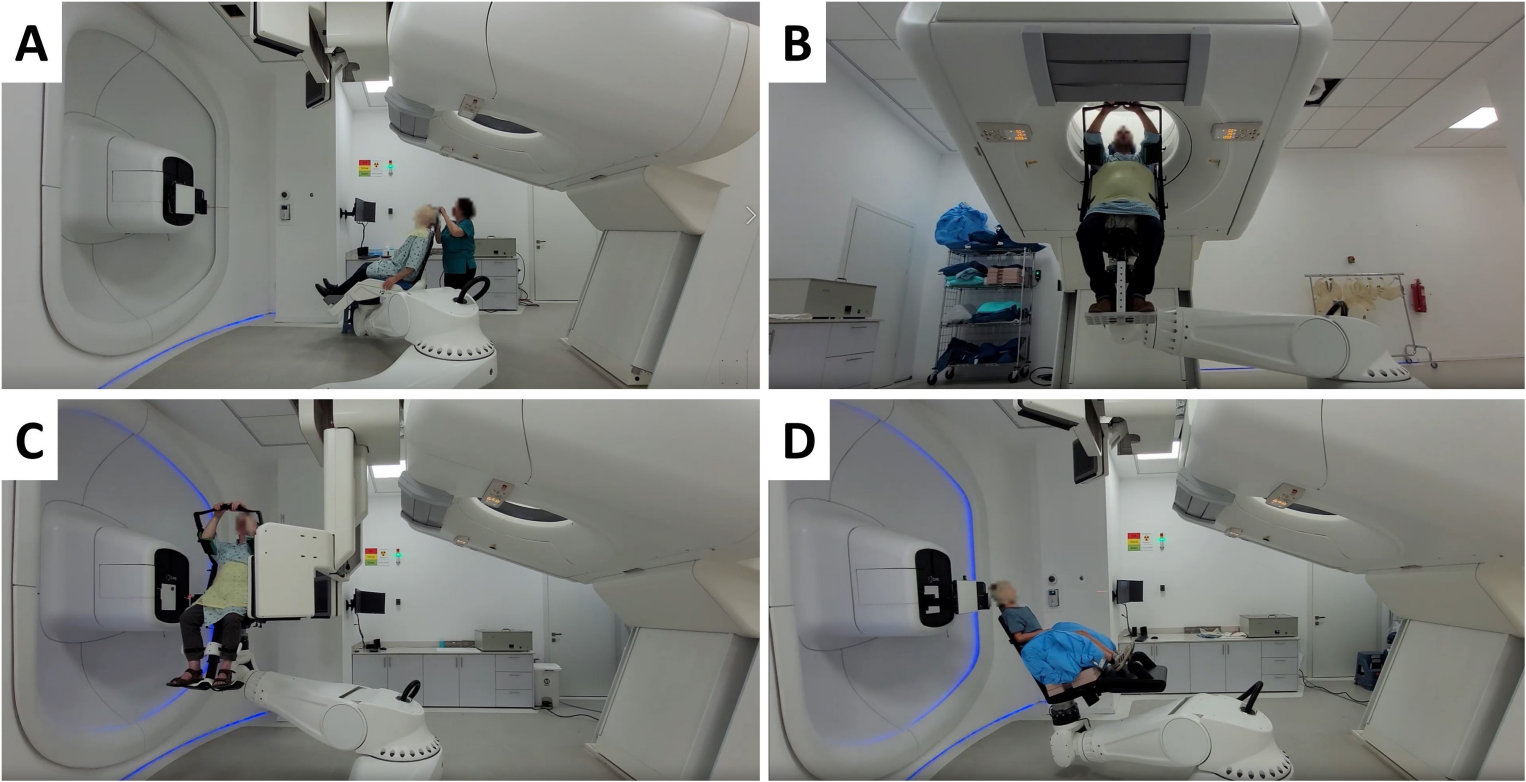
Different P-ARTIS positions and components for patient-specific cancer sites: **(A)** Patient loading and immobilization for head and neck cancer. **(B)** Imaging for treatment planning for liver case. **(C)** Patient position verification at the treatment isocenter for prostate cancer. **(D)** Treatment with range shifter for pediatric head and neck sites.

P-ARTIS supports predetermined positions relative to the room coordinate system as illustrated in **Figure 4**: (4A) loading position to immobilize the patient in the chair, (4B) imaging position for vertical CT, (4C and 4D) treatment position at isocenter during proton irradiation. Adjustment of the patient position is performed based on image registration results, utilizing a 3D/3D correction vector from planning CT/treatment CT image registration and a 2D/3D correction vector from planning CT/X-ray radiography image registration. In addition, there are robot positions related to dosimetry procedures with tools like the IBA Lynx, the IBA MatriXX 2D array of ionization chambers and the PTW MP3 water phantom.

### 2.5 Clinical CT commissioning

P-ARTIS CT commissioning was performed using a mass density calibrated Gammex tissue characterization phantom model 467. The phantom was scanned three times, alternating the rods to cover all available materials in the desired array. One rod of each material was positioned in the inner and outer radius of the phantom on opposite sides, making the density tissue replacement rods evenly distributed around the scans (33). For each rod material, 1 cm diameter regions of interest (ROIs) were used at three different slice heights. The final Hounsfield Units (HU) of each material was obtained by averaging all ROI measurements for each material.

### 2.6 External dosimetry

#### 2.6.1 Beam spot profiles

**Figure 5:**
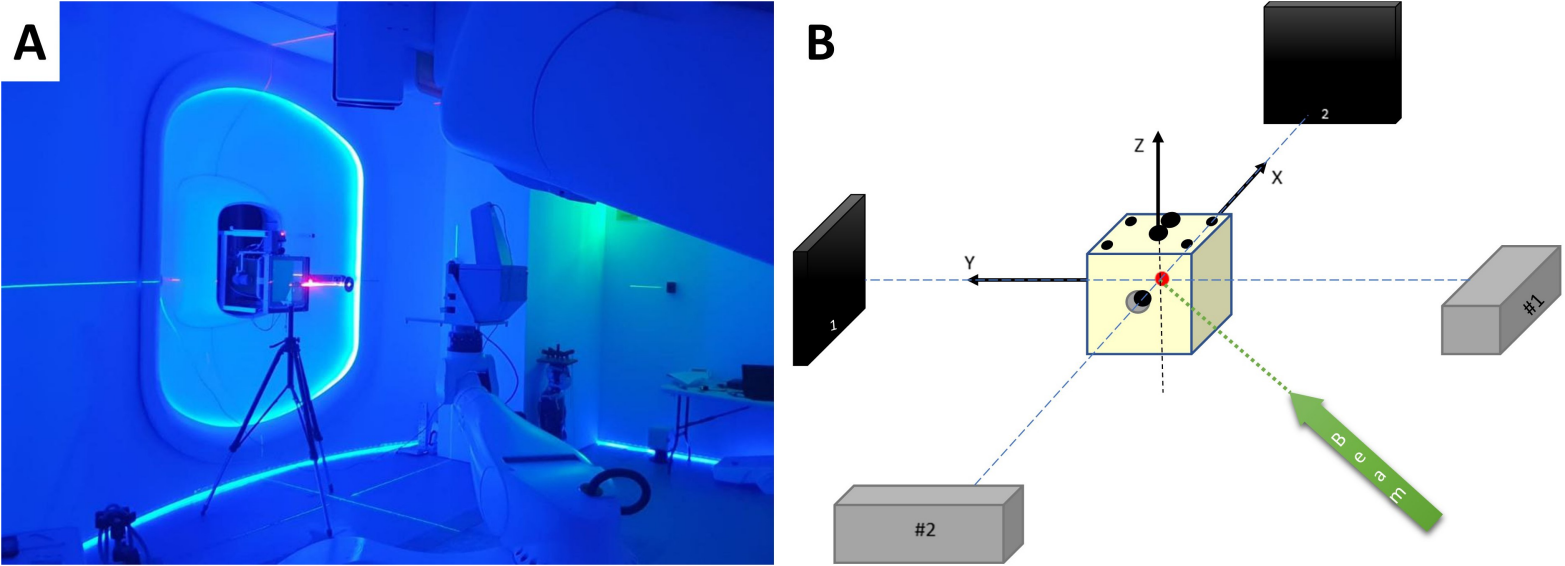
**(A)** Primary P-ARTIS robot calibration based on Lynx beam position readings. **(B)** Schematic view of the 2D X-ray system calibration.

The magnetic beam optics was adjusted and optimized using the beam visualization system (A9) situated in the nozzle. The system measures the beam profile with an accuracy and precision of ±0.1 mm at a fast rate of 300 fps, enabling rapid and efficient beam parameter monitoring during concurrent operation. In addition, the Lynx detector with an active detection area of 30 cm × 30 cm and a spatial resolution of 0.5 mm has been used to measure the beam profile and position at the isocenter for multiple energies at 10 MeV intervals for commissioning and ongoing quality assurance (QA) studies (**Figure 5A**). The detector was aligned with the P-ARTIS reference system axes, and commissioned for linearity, response reproducibility and homogeneity, iris aperture response dependence, and geometric distortion (34, 36). Secondary validation of the Lynx measurements was performed by comparing them with EBT3 film (Gafchromic, Bridgewater, NJ, USA) measurements for selected beam energies.

The feasibility of modeling the physical spot with a bivariate normal distribution was checked (37). The ellipticity, η was calculated using the two sigma values of a 2D Gaussian fit according to Equation 1:


(1) $$\eta =\frac{\sigma_{max}-\sigma_{min}}{\sigma_{max}}\;$$

where $\sigma_{max}\;$ and $\sigma_{min}$ were the larger and smaller sigma values of the 2D Gaussian profile, respectively.

#### 2.6.2 Integrated depth-dose curves

Two parallel plate ionization chambers, PTW Bragg Peak Chamber 34070 and 34080, in combination with a calibrated PTW Tandem XDR electrometer and a PTW MP3 water tank were used to measure the IDDs of monoenergetic pencil beams. The homogeneity of the chamber response was verified by aiming the same beam spot at various locations within the chamber (38). Additionally, the linearity of the ionization chamber response was confirmed, and the chamber polarity and ion recombination effects were assessed across different depths and energy levels.

An alignment QA check with the water tank and both chambers was performed prior to measuring the IDDs. The overall reproducibility of the system was evaluated by repeating each individual energy setup and IDD measurement nine times and calculating the mean and standard deviation of the distal R_90_ values for levels from 70 to 250 MeV, every 10 MeV. In addition, the consistency between the IDD data and the corresponding beam energies entered the treatment planning system (TPS) was manually checked by verifying that these energies were within tolerance of the energy values independently provided by the DMS during the IDD acquisition.

For energies 70 - 80 MeV the Bragg peak section was scanned with a resolution of 0.2 mm and for 90 - 250 MeV with a resolution of 0.3 mm. The post-peak region was scanned with a resolution of 0.5 mm to 2 mm and the build-up regions were scanned with a resolution of 1-5 mm.

#### 2.6.3 Absolute dose measurements

Absolute dose values were measured using a parallel plate ionization chamber and an electrometer (PTW Roos 34001, UNIDOS Webline) in a field of 10 cm × 10 cm for each energy. The chamber and electrometer were calibrated in terms of the N_D,W_ in ^60^Co at the Radiation Control Unit of Israeli Ministry of Health. The following standard coefficients were calculated using the IAEA TRS-398 formalism (40): *K_s_*, factor to correct the response of an ionization chamber for the lack of complete charge collection (Equation 2), *K_pol_*, factor to correct the response of an ionization chamber for the effect of a change in polarity (Equation 3), *K_TP_*, factor to correct the ionization chamber response for ambient temperature and pressure (Equation 4). Recombination, polarity, temperature, and pressure factors were calculated for ionization chamber voltages of ±300V and +100V:


(2) $$K_{s}=\alpha_{0}+\alpha_{2}\frac{M_{+300}}{M_{+100}}+\alpha_{3}{\left(\frac{M_{+300}}{M_{+100}}\right)}^{2}\;$$

(3) $$K_{pol}=\frac{\left|M_{+300}\right|+\left|M_{-300}\right|}{2M_{+300}}\;$$

(4) $$K_{TP}=\frac{\left(273.2+T\right)P_{0}}{\left(273.2+T_{0}\right)P}$$

The coefficients $\alpha_{0},\;\alpha_{2},\;\alpha_{3}$ in the Equation 2 (“two-voltage” technique) were used as for the pulsed-scanned radiation following the TRS-398 protocol (40). In Equations 2-4 *M_voltage_, T, P* correspond to readings from the ionization chamber at a certain voltage, temperature sensor, and pressure sensor, respectively, $T_{0}=20{}^{\circ}C$ and $P_{0}=1013.25\;kPa$ . All measurements were performed in the PTW MP3 water tank at water equivalent depths ranging from 19.9 mm (for 70 MeV) to 150.0 mm (for 250 MeV).

The method for determining the reference dose value was based on the procedure described in TRS-398 (40). The commissioned TPS was used to generate plans for the homogeneous irradiation of the 10 cm × 10 cm × 10 cm cubes placed inside the water phantom. The centers of the cubes were placed at depths of 6 cm, 15 cm and 19 cm inside the water phantom. The resulting dose was measured at a depth of 15 cm using a PTW Farmer chamber. The measurements were corrected using tabulated kQ values from TRS-398 (40) determined for SOBP proton beams and a specific ionization chamber. The linearity of the Farmer ionization chamber response was confirmed, and polarity and recombination corrections were established for the described SOBP fields.

### 2.7 End-to-end testing

The P-ARTIS PPS calibration for the treatment isocenter was conducted following BDS calibration. The Lynx detector was mounted on the robot as shown in **Figure 5A**. The robot-specific coordinate system was established using in-house software based on three parallel surfaces located at 450 mm, 700 mm, and 950 mm from the nozzle. This was guided by BDS lasers and room reference points. Five points with coordinates (0, 0), (100, 100), (100, -100), (-100, 100), (-100, -100) mm were shot on each surface to construct a 3D coordinate system. The positioning of the retractable X-ray detectors and subsequent robot rotation was calibrated using a solid water cube phantom with radiopaque inserts relative to the BDS and room lasers. The accuracy and repeatability of the PPS were measured and confirmed using the Radian PLUS laser tracker (Microservice SRL, Turin, Italy) positioned in the treatment room, with an accuracy of 0.3 mm. Calibration of the 2D imaging system involved aligning the phantom center marker with the image center, applying X and Y corrections based on the marker location, adjusting the detector position, considering a pixel size of 0.19 mm. Symmetric images for ±45° were verified, ensuring alignment of the source-detector pairs and confirming equal distances from the isocenter, as shown schematically in **Figure 5B**. CT-to-robot calibration was then performed using the same cube phantom placed in the imaging isocenter and the OncoBrain CT acquisition mode.

The RayStation version 10B (RaySearch Laboratories AB, Stockholm, Sweden) was used for beam modeling and treatment planning. Development of RayStation beam model required input from IDDs, spot profiles, and absolute dose values for a set of energies from 70 MeV to 250 MeV spaced by 10 MeV (39). The version of RayStation TPS currently used by the center assumes a symmetric 2D beam profile approximation ( $\sigma_{x}=\sigma_{y}$ ). The symmetric beam profile assumption was verified at the time of commissioning and is now part of the ongoing medical physics periodic QA. The absolute dose measurements with a Farmer chamber at the center of SOBP curves and several points along the central beam axis for different field sizes, SOBP widths, and depths were conducted to verify the accuracy of the RayStation TPS model. A Farmer chamber was chosen for these validation tests based on the TRS-398 recommendations (40).

**Figure 6:**
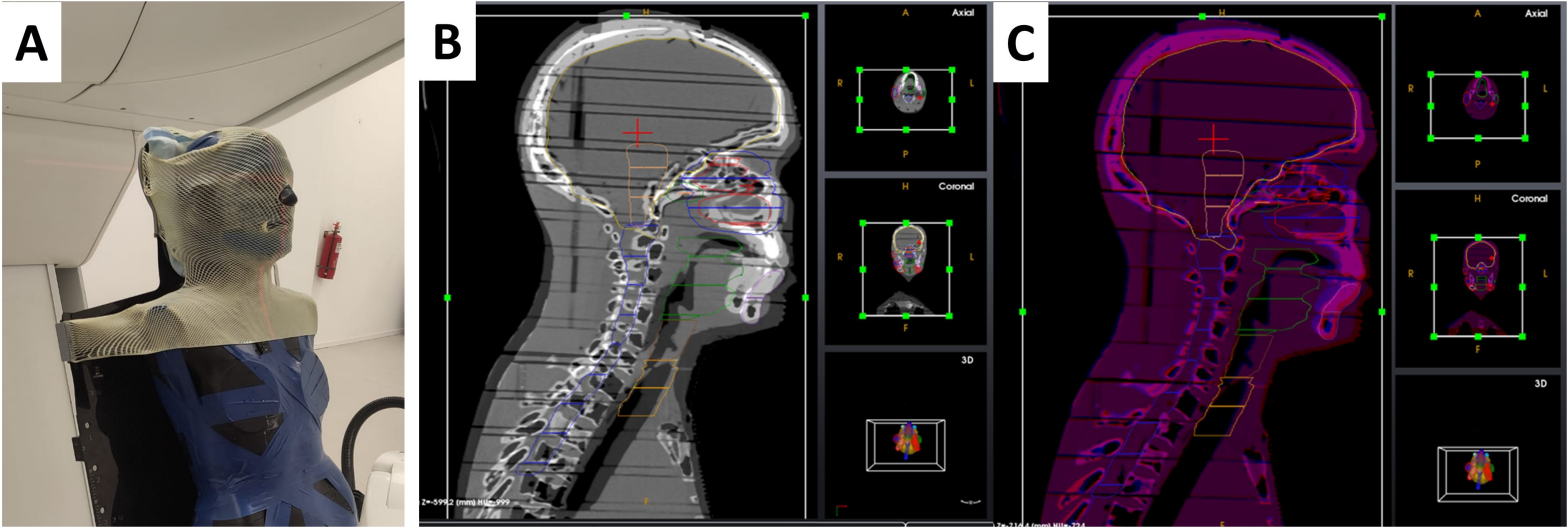
Patient positioning and imaging system calibration and setup for end-to-end testing. **(A)** CT image acquisition setup for head and neck case. **(B, C)** 3D/3D registration of the head and neck area before **(B)** and after **(C)** alignment.

**Figure 7:**
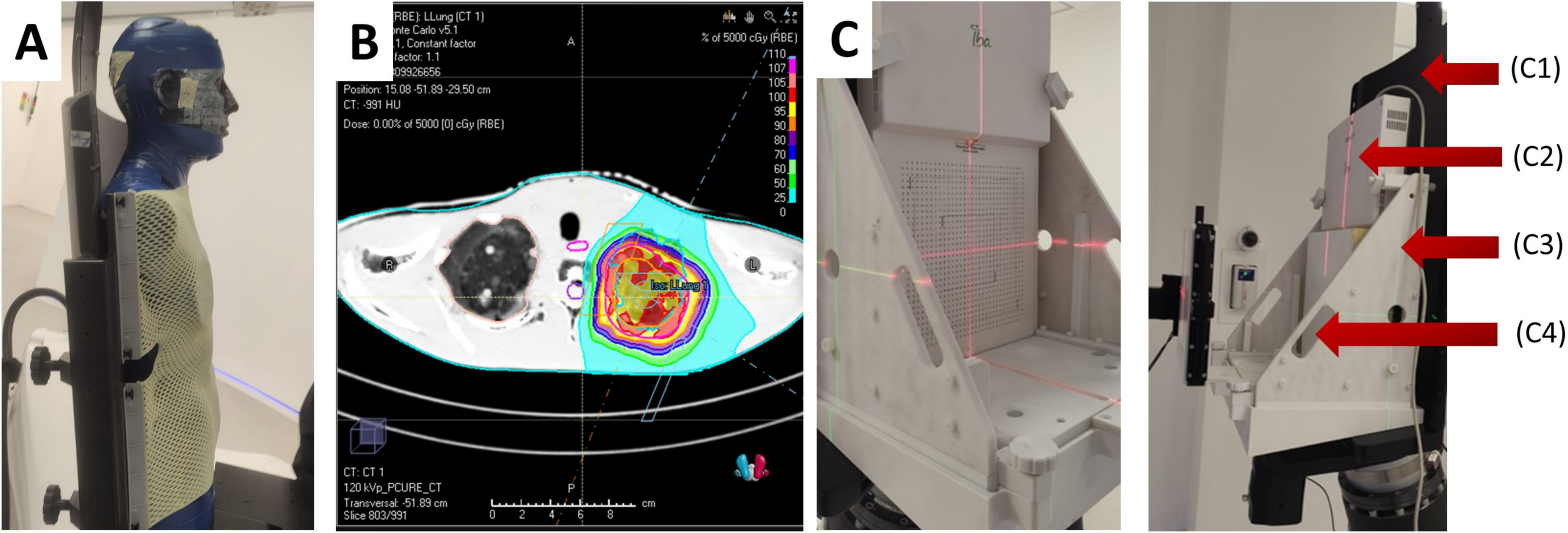
**(A)** CT image acquisition setup for thorax simulations with anthropomorphic phantom. **(B)** Example of dose calculation for the left lung of phantom geometry. **(C)** Mounted MatriXX assembly without (left) and with (right) solid water, where C1 – chair, C2 – IBA MatriXX, C3 – holder, C4 – solid water.

During the treatment planning simulation, the anthropomorphic phantom was loaded into the PPS. The phantom was immobilized using a 5-point thermoplastic mask (Orfit, Wijnegem, Belgium) for central nervous system (CNS) or head and neck (H&N) treatment sites (**Figure 6A**) and a torso thermoplastic mask for thoracic sites (**Figure 7A**). The preset OncoBrain and Thorax protocols with 0.45 mm and 0.9 mm slice thickness were chosen for H&N, and thorax CT acquisition, respectively.

The positioning of the phantom involved locating the center of the cranium (CNS), the level of the C4 vertebrae (H&N), and the center of the thorax along the spinal cord (thorax). Geometric evaluation prior to treatment planning was performed by monitoring the residual positioning error calculated by the 3D/3D and 2D/3D registration software using images of the anthropomorphic phantom. Positioning accuracy is monitored as a 3D shift between the desired phantom position as defined by the treatment plan and the actual position as measured by the 2D/3D image registration. The 3D shift is defined as:


(5) $$D=\sqrt{\text{Δ}x^{2}+\text{Δ}y^{2}+\text{Δ}z^{2}}$$

The phantom underwent additional CT scans from the crown through T6 (CNS/H&N) as shown in **Figures 6B, C**, and from the base of the skull (C1) through T12 (thorax). The acquired CT images were used for 3D/3D registration with a simulation CT dataset providing a 6-DoF correction vector. The 3D/3D correction vector was applied during PPS motion to the first treatment field, subsequently verified by 2D/3D registration using 2D X-ray images and digitally reconstructed radiographs (DRRs) generated from the reference CT. Geometrical acceptance criteria included an initial correction Euclidean vector below 10 mm and a residual shift (Euclidean correction vector calculated after the applied shift) below 2 mm for treatment isocenter.

All treatment plans were simulated using RayStation Monte Carlo (MC) algorithm. 10 clinical scenarios were selected for the comparison, exemplified in **Figure 7B**. The plans were dosimetrically verified using radiochromic films and the IBA MatriXX PT. The Gamma analysis was performed with 3%/3mm pass criteria.

To account for the incompatibility of the MatriXX with the anthropomorphic phantom geometry, a custom QA phantom was used, consisting of solid water and the MatriXX, as shown in **Figure 7C**. The position of the MatriXX within the phantom was adjusted by offsetting the solid water plates proximally to align with the desired measurement plane. The QA phantom underwent a CT scan, and the CT data was imported into the TPS. A 20 cm × 20 cm × 10 cm field was used for detector calibration, with the measured dose set to 100 cGy. Four simple geometry plans were run for dose and spatial distribution evaluation. The MyQA software platform provided with the MatriXX PT was used for reporting and gamma index calculation. Finally, treatment plans were recalculated to the QA phantom geometry, executed, and reviewed using the same pass criteria.

## 3 Results

### 3.1 Clinical CT commissioning

The results of CT HU to mass density conversion are demonstrated in **Table 1**. The imported mass density of each rod tissue was associated with a material elemental composition and mean ionization energy, and then correlated it to stopping power.

**Table 1:** Results of CT Hounsfield Units to mass density conversion.

Tissue Substitute Material	HU	Mass density (g/cm^3^)
SB3 Cortical Bone	1194.167	1.821
CB2-50% CaCO3	781.5667	1.558
CB2-30% CaCO3	424.1833	1.333
B200 Bone Mineral	190.5667	1.152
IB Inner Bone	180.7167	1.143
LV1 Liver	74.76667	1.091
BRN-SR2 Brain	23.95	1.05
Water	1	1
CT Solid Water	-0.9	1.015
BR-12 Breast	-42.35	0.983
AP6 Adipose	-90.3667	0.948
Lung LN-450	-532.1	0.47
Lung LN-300	-714.7	0.28
Air	-988.54	0

### 3.2 Extracted beam time structure and dose monitoring system calibration

**Figure 8:**
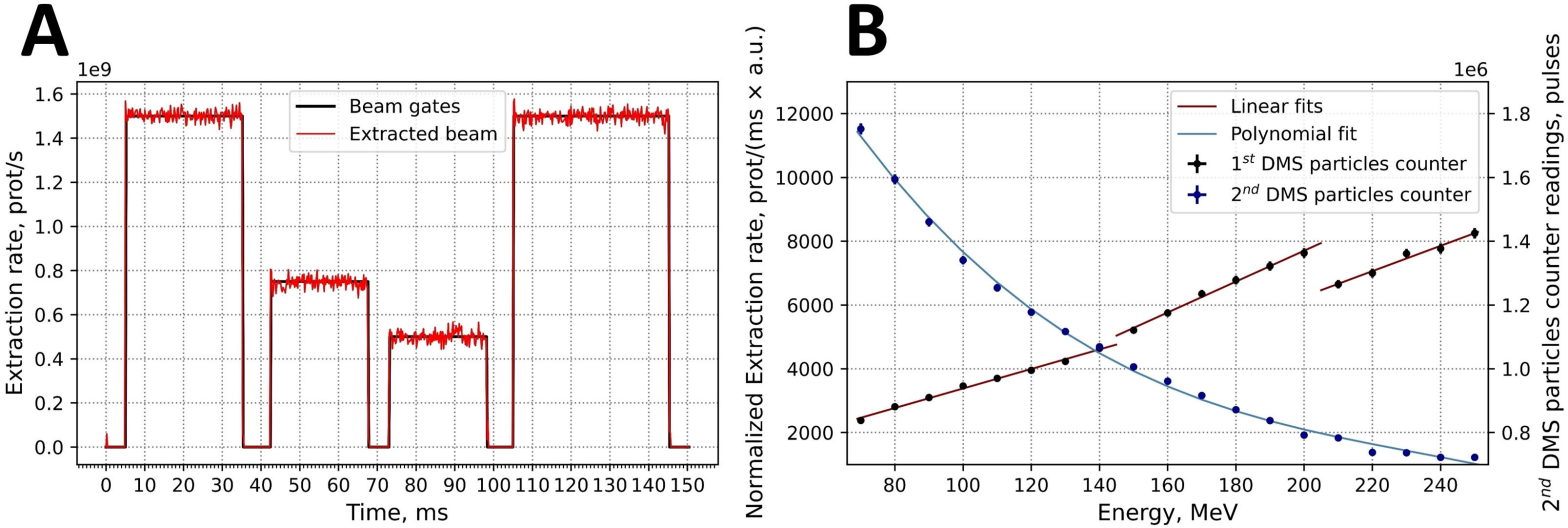
**(A)** Example for the time structure of an intensity- and time-modulated 150.0 MeV proton beam. **(B)** Calibration curves for both built-in DMS particle number counters: 1^st^ DMS particle counter in normalized extraction rate values and 2^nd^ DMS particle counter in pulse values.

The time structure of the extracted beam was continuously monitored by the integrated BCMs of the BDS, an example of which is shown in **Figure 8A**. The minimum planned spot length and the time between two consecutive spots were both 5 ms. This time was chosen to optimize the stability of the extraction. Based on readings every 0.2 ms the deviations of the extracted intensity (*I*) can be relatively high within one spot *(* $I_{max}\;-\;I_{min}$ *)/* $I_{plan}$ up to 50%. However, the accelerator server constantly checks the extracted beam intensity in a real-time feedback mode and generates spots with a length of less than 5 ms if the extracted beam intensity exceeds the planned value. This feedback system allowed to achieve the target level of deviation $\left|I_{delivered}\;-\;I_{plan}\right|$ */* $I_{plan}$ ) <2% for all the registered scenarios. The technical limit for the spot length was found to be 0.6 ms (time to start the extraction + time to stabilize the extraction + time to stop the extraction), since the minimum time to change the state of the accelerator system is 0.2 ms. The current accelerator configuration allows working with maximum extraction rates of 7.3×10^8^ protons/second at 70 MeV and 2.4×10^9^ protons/second at 250 MeV, with a linear dependence of intensity rate on energy between these two points.

The calibration of the two DMS-associated particle number counters for beam intensity are based on the readings of external Faraday cup. The first counter is calibrated daily (**Figure 8B**, red lines). Three separate linear fits to the measured points were generated according to the three extraction targets that are used for different energy intervals. The first target was used up to energies of 149.9 MeV and the second target up to 204.9 MeV. The second counter was calibrated once. In this data, was fitted by the polynomial relationship (Equation 6):


(6) $$P=-1.52\times 10^{-1}E^{3}+1.06\times 10^{2}E^{2}-2.68\times 10^{5}E+3.13\times 10^{6}$$

where *P* was the number of pulses and *E* is energy in MeV (**Figure 4B**). Each pulse corresponds to a particle charge of 0.005 nC.

### 3.3 Integrated depth-dose curves

**Figure 9:**
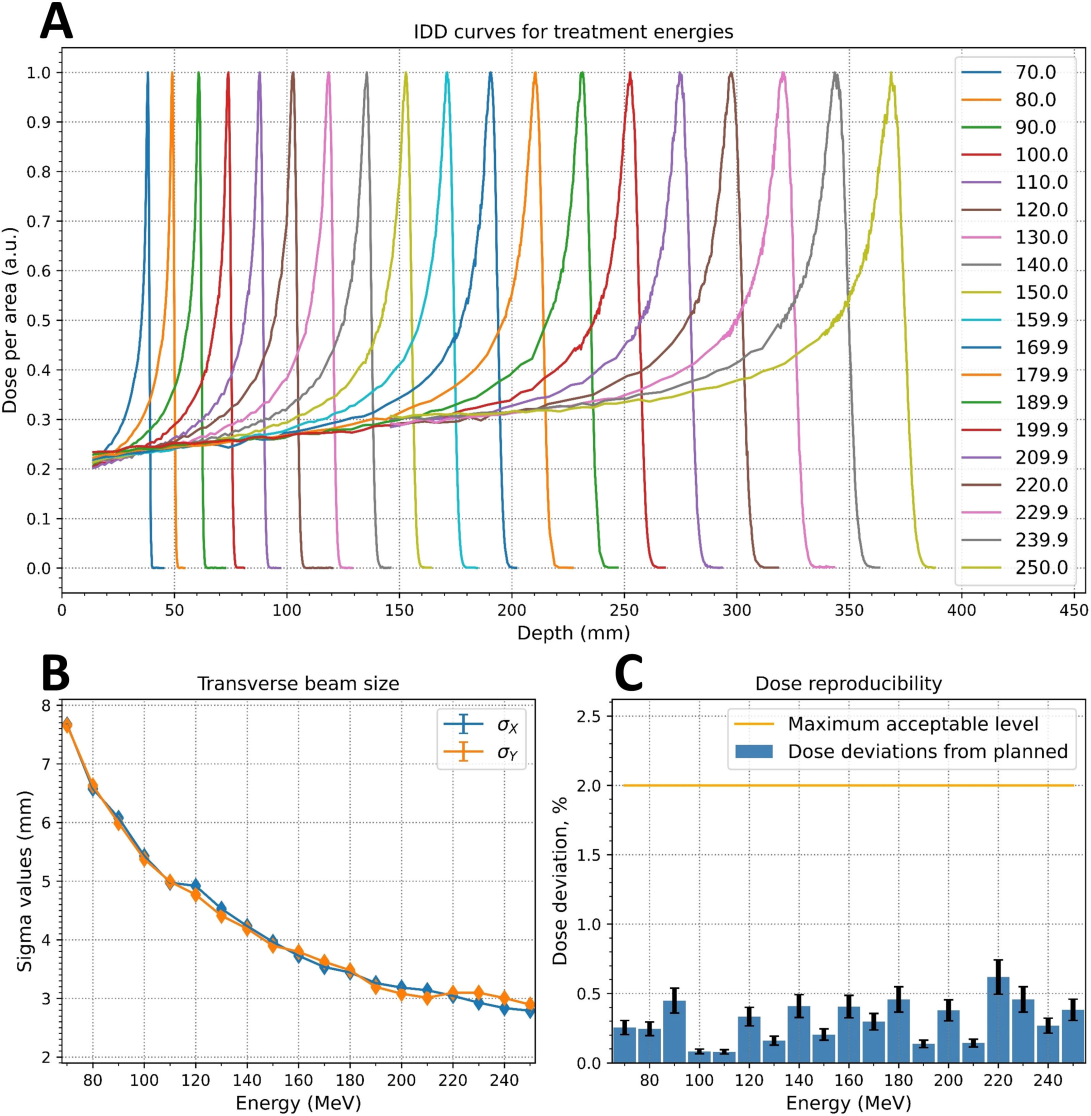
Measured critical beam parameters: **(A)** integrated depth-dose (IDD) curves; **(B)** energy dependence of the beam spot sigma values at irradiation isocenter; **(C)** dose reproducibility measurements.

**Figure 9A** shows normalized IDDs that were loaded into the TPS. Energy accuracy was ±0.1 MeV for all cases based on accelerator RF measurements. The determination of distal R_90_ values was based on the measured IDDs, and the system reproducibility was evaluated by confirming that the ranges were within ±0.1 mm for all cases.

### 3.4 Beam spot profiles

The ellipticity values, η (Equation 1), were less than 0.057 for the Lynx and EBT3 measurements. **Table 2** displays the measured sigma values for 5 of the 19 energy levels analyzed with the IBA myQA software. The dependence of the sigma values for the remaining energies was monotonically decreasing, as shown in **Figure 9B** for the Lynx placed at the irradiation isocenter (700 mm from the nozzle).

**Table 2:** Proton beam profile sigma values (mm) in horizontal (x) and vertical (y) direction at various distances from the edge of the nozzle.

Distance (cm) Energy (MeV)	45		55		70		85		100	
	σ_x_	σ_y_	σ_x_	σ_y_	σ_x_	σ_y_	σ_x_	σ_y_	σ_x_	σ_y_
70	6.16 ± 0.08	6.06 ± 0.08	6.73 ± 0.07	6.66 ± 0.07	7.67 ± 0.04	7.66 ± 0.04	8.71 ± 0.05	8.76 ± 0.05	9.77 ± 0.06	9.87 ± 0.06
100	4.30 ± 0.07	4.16 ± 0.07	4.71 ± 0.06	4.61 ± 0.06	5.43 ± 0.04	5.38 ± 0.04	6.17 ± 0.03	6.16 ± 0.03	6.92 ± 0.04	7.01 ± 0.04
150	3.21 ± 0.07	3.07 ± 0.07	3.49 ± 0.05	3.39 ± 0.05	3.96 ± 0.03	3.90 ± 0.03	4.46 ± 0.01	4.46 ± 0.01	4.99 ± 0.02	5.03 ± 0.02
200	2.60 ± 0.07	2.47 ± 0.07	2.81 ± 0.06	2.69 ± 0.06	3.18 ± 0.05	3.08 ± 0.05	3.53 ± 0.03	3.49 ± 0.03	3.97 ± 0.03	3.95 ± 0.03
250	2.34 ± 0.06	2.44 ± 0.06	2.5 ± 0.1	2.7 ± 0.1	2.79 ± 0.09	2.89 ± 0.09	3.1 ± 0.1	3.2 ± 0.1	3.5 ± 0.2	3.7 ± 0.2

### 3.5 Absolute dose values

Absolute dose measurements were performed 3 times for each energy and ionization chamber voltage. The total amount of protons was 1.681×10^11^ per plan. Correction factors for absolute dose calculation are shown below (Equations 7-10):


(7) $$0.999\;\leq \;K_{s}\;\leq 1.019\;$$

(8) $$0.993\;\leq \;K_{pol}\;\leq 1.010\;$$

(9) $$1.015\;\leq \;K_{TP}\;\leq 1.019\;$$

(10) $$1.000\;\leq \;K_{QQ0}\;\leq 1.002\;$$

For all measured energies, the maximum deviation between measured and planned dose was less than 0.6%, as shown in **Figure 9C**. The deviation of the DMS two proton count readings from the planned dose was less than 1% for all energies except for 250 MeV, where the deviation was slightly below than 2%.

### 3.6 End-to-end testing

To test the performance of the system in terms of image-guided positioning and treatment plan compliance, the full image-guided scenario was performed. Five H&N and five thoracic cases were evaluated for the ability of the described PT system to deliver treatment plans in terms of positioning and dosimetric accuracy. This end-to-end test involved the coordination of all subsystems of the facility and involved third-party dosimetric and treatment planning equipment. The results of the end-to-end test are presented in **Table 3**.

**Table 3:** Accuracy of the image guided positioning at isocenter following 3D/3D registration, monitored by subsequent 2D/3D image registration and Gamma analysis comparing planned and measured doses for standard treatment scenarios.

Region	Site	Initial upper confidence interval (mm)	Number of image-guided positioning iterations (2D/3D registration) at isocenter and the size of corresponding field (number, cm^2^)				Residual upper confidence interval (mm)	Target dose (cGy)	Number of fractions	Number of repetitions	Gamma index (3%, 3 mm) per plan (%)		
			Field 1	Field 2	Field 3	Field 4					Min	Median	Max
CNS	L Parietal	7.65	1, 7 × 7	1, 7 × 7	NA	NA	0.560	6000	30	3	100	100	100
	Base of Skull	1.36	1, 4 × 5	1, 4 × 5	NA	NA	0.29	6000	30	3	98.9	100	100
Head	Nasopharynx	1.30	1, 5 × 7	1, 7 × 9	1, 7 × 9	NA	0.34	5400	30	3	95.3	100	100
	R Parotid	2.33	1, 9 × 9	1, 9 × 9	NA	NA	0.51	5400	30	3	100	100	100
Neck	Bilateral Neck (Right, Left)	1.46	1, 13 × 11	1, 11 × 11	1, 11 × 11	1, 13 × 11	0.40	5400, 6300	30	3	96	98	100
Thorax	Mediastinum	3.08	1, 7 × 10	1, 7 × 10	NA	NA	0.34	6000	20	3	96.3	98.8	100
	R Chest Wall	5.25	1, 7 × 10	1, 7 × 9	NA	NA	0.74	5000	20	3	100	100	100
	Esophagus	2.90	1, 8 × 10	1, 8 × 12	1, 8 × 11	NA	0.49	4500	25	3	95.4	95.4	95.4
	L Upper Lung	4.29	1, 9 × 7	1, 9 × 7	1, 9 × 7	NA	1.70	5000	5	3	100	100	100
	R Lower Lung	4.89	1, 9 × 8	1, 10 × 10	NA	NA	0.38	5000	10	3	100	100	100

As shown, for all 10 plans, the initial laser-free 3D/3D image-guided positioning brought the anthropomorphic phantom to the isocenter well below the defined threshold of 10 mm Euclidean displacement (and within ±5 mm in absolute values). Additional 2D/3D image-guided positioning of the phantom at the isocenter for each successive field of the plan resulted in sub-millimeter accuracy (defined acceptance criteria: <2 mm Euclidean displacement). All absolute displacement values were within ±1 mm. A single 2D/3D image registration event was required to correct the positional shift in all cases.

The TPS dose calculation model was verified using these ten plans. The gamma analysis pass rate (3%, 3 mm) was greater than 95% for all scenarios. The overall confidence interval (CI) was 0.98-1 (lower bound exceeds 0.95). In the mixed effect model, the effect was statistically significant (p=0.04), with a CI of 0.98-1 for H&N and 0.96-0.99 for thorax. Both CIs exceed 0.95, the values for H&N are significantly higher than those for thorax.

## 4 Discussion

Recent trends in the PT market show increased interest in compact, single-room PT centers (41). 22 out of 27 PT facilities under construction and installed by IBA SA fir the last four years are single-room units (42). However, the use of cyclotrons for beam acceleration requires the installation of two shielded vaults, effectively separating the accelerator from the patient. In addition, the treatment room equipment, including gantries, is designed to occupy three-story vaults.

Synchrotrons and pencil beam scanning have very low levels of non-primary radiation background and do not require internal shielding concrete between the accelerator and the patient (20). Replacing cyclotrons with compact synchrotrons (e.g. P-Cure or Protom synchrotrons) further reduces the footprint of the PT facility by allowing the accelerator and image-guided positioning system to be installed in the same treatment vault. Replacing a gantry-based beam delivery with a gantry-less configuration provides an additional opportunity to reduce the treatment room height to a single floor dimension.

The similar approach of placing the accelerator and the upright patient positioning system in the same vault has recently been claimed (but using synchrocyclotrons instead of synchrotrons) by the companies Mevion and Leo Cancer Care with their joint Mevion Fit system. The first system is currently under construction (41).

The required vault dimensions for the commissioned system are 12×7.5×4 m^3^ and can be further reduced based on clinical requirements. These dimensions allow for integration into existing vaults. Due to synchrotron modular design (as opposed to the cyclotron), it can be installed in a vault without requiring additional space or moving to other treatment rooms. The maximum weight of the non-separable module of this synchrotron is less than 450 kg, which makes it possible to use non-specialized means of equipment delivery and installation. The comparatively low weight of the equipment allows the synchrotron to be tilted relative to the floor, saving additional space, but significantly increasing the complexity and potentially the cost of the installation.

The flexibility and modularity of the facility described offers potential for future expansion and further optimization. Although the current facility is operational and used for patient treatment, there are three key areas for potential future upgrades. Firstly, an increase in beam energy up to 330 MeV and implementation of an ultra-low intensity extraction mode for proton radiography and CT could be explored, as this capability was demonstrated for the similar synchrotron (43). Secondly, work is currently underway to enable stepwise beam acceleration within one cycle for the synchrotron, which could lead to the extraction of multiple beam energies in one acceleration cycle and thus reduction in irradiation time by more than factor of two (32). Thirdly, to facilitate the extraction of protons of several energies and achieve the required dose rates for proton FLASH therapy for Bragg peak region, which currently is in first clinical trials but for transmission proton beam (44), a modernized beam injection system is being developed to increase the beam current captured and subsequently extracted while maintaining the extraction rate. Lastly, mini-beam proton therapy has also shown promising clinical results with sparing of normal tissue (45). Furthermore, as part of the current research on mini-beams, we plan to fabricate and implement a collimator array for mini-beam delivery without the need for structural beamline modifications. This collimator array will be attached to the beam delivery system instead of the range shifter and will move into the beam during treatment.

The dosimetric and geometric validation of the commissioned facility demonstrated the clinical quality of treatment plans generated for this gantry-less PT system using anthropomorphic phantom positioned on a chair. Currently, clinical trials are underway to provide additional supportive clinical data in the treatment of cranial, head and neck, thoracic and pancreatic patients seated with a gantry-less system (46).

## 5 Conclusion

A comprehensive commissioning of a modern single-room proton therapy facility has been conducted. The facility is based on a compact proton synchrotron and a novel patient positioning system that can accommodate the patient in upright positions. The tests demonstrated high robustness of the entire system particularly in terms of high accuracy of the robotic arm, the image registration performances and high pass rates of Gamma index checks for delivered dose plans. After commissioning, the facility started to treat patients in March 2023. It is the first proton therapy facility in Israel and the entire Middle East region. Future upgrades are planned in terms of increasing the beam energy and intensity, multi-energy extraction per cycle.

## Data Availability

The raw data supporting the conclusions of this article will be made available by the authors, without undue reservation.
